# Vagus Nerve Stimulation Alters Phase Synchrony of the Anterior Cingulate Cortex and Facilitates Decision Making in Rats

**DOI:** 10.1038/srep35135

**Published:** 2016-10-12

**Authors:** Bing Cao, Jun Wang, Mahadi Shahed, Beth Jelfs, Rosa H. M. Chan, Ying Li

**Affiliations:** 1 Department of Biomedical Sciences, City University of Hong Kong, Tat Chee Avenue, Kowloon, Hong Kong; 2 Centre for Biosystems, Neuroscience, and Nanotechnology, City University of Hong Kong, Kowloon, Hong Kong; 3 Department of Electronic Engineering, City University of Hong Kong, Kowloon, Hong Kong; 4 Shenzhen Key Lab of Neuropsychiatric Modulation, CAS Center for Excellence in Brain Science, Shenzhen Institutes of Advanced Technology, Chinese Academy of Sciences, Shenzhen 518055, Hong Kong; 5 School of Veterinary Medicine, City University of Hong Kong, Kowloon, Hong Kong

## Abstract

Vagus nerve stimulation (VNS) can enhance memory and cognitive functions in both rats and humans. Studies have shown that VNS influenced decision-making in epileptic patients. However, the sites of action involved in the cognitive-enhancement are poorly understood. By employing a conscious rat model equipped with vagus nerve cuff electrode, we assess the role of chronic VNS on decision-making in rat gambling task (RGT). Simultaneous multichannel-recordings offer an ideal setup to test the hypothesis that VNS may induce alterations of in both spike-field-coherence and synchronization of theta oscillations across brain areas in the anterior cingulate cortex (ACC) and basolateral amygdala (BLA). Daily VNS, administered immediately following training sessions of RGT, caused an increase in ‘good decision-maker’ rats. Neural spikes in the ACC became synchronized with the ongoing theta oscillations of local field potential (LFP) in BLA following VNS. Moreover, cross-correlation analysis revealed synchronization between the ACC and BLA. Our results provide specific evidence that VNS facilitates decision-making and unveils several important roles for VNS in regulating LFP and spike phases, as well as enhancing spike-phase coherence between key brain areas involved in cognitive performance. These data may serve to provide fundamental notions regarding neurophysiological biomarkers for therapeutic VNS in cognitive impairment.

The viscero-sensation is ‘a faculty of perception that does not depend upon any outward sense’^1^, but which acts to influence the elicited behavioral response. Clinically, vagus nerve stimulation (VNS) is used to treat refractory epilepsy^2^,^3^, and resistant depression^4^. VNS has also shown several beneficial effects for mood enhancement^2^, improved cognitive abilities in Alzheimer’s patients^5^, or reducing edema following brain trauma^6^. Because of the known release of multiple neuromodulators, VNS has recently become an object of study in regulating cortical plasticity^7^. Clark *et al*. have demonstrated that VNS, administered immediately after training, enhanced memory retention performance for an inhibitory-avoidance task in rats^8^, and enhanced recognition in human subjects^9^. We have previously explored the visceral analgesic properties of sub-diaphragmatic VNS in rats and showed that low-intensity VNS activates vagal afferent A∂ fibers to reduce visceral pain^10^. Recently, we have shown that visceral pain-related affective memory was facilitated by VNS, underscoring the importance of memory in visceral pain perception^11^.

Making a decision under complicated and uncertain conditions is a basic cognitive process for adaption relying on the integration of several executive functions. In humans, decision-making has been accurately modeled in a laboratory setting using the Iowa gambling task (IGT)^12^. A human experimental study by Martin *et al*.^13^ investigated the effect of VNS on decision-making. Improved performances were shown in participants but despite this the underlying mechanisms are unknown. Recently, we have conducted a decision-making test - the rat gambling task (RGT)- and found that, similar to the findings of the most advantageous strategy from the IGT utilized in human studies^14^, most rats can learn to maximize their food reward by reasoning. Thus, during the RGT preferring the more profitable options associated with smaller immediate gain but lower risk of punishment (time-out)^15^. Of note, no animal models have been developed to date to examine the effects of chronic VNS on decision-making.

Studies of brain-lesion and psychiatric patients have discovered the specific prefrontal cortex (PFC) areas mediating decision-making^16^,^17^. Recent animal studies have shown that decision-making performances in the RGT depend on the integrated function of several sub-regions of the PFC, especially the prelimbic, cingulated and orbitofrontal cortices, and amygdala^18^. Several imaging studies of VNS, particularly those using fMRI, have found changes in the PFC, orbitofrontal cortex, insula, anterior temporal poles and the hypothalamus^19^,^20^. These data suggest that VNS may be affecting areas of the brain involved in decision-making. In the current study we evaluated the effects of VNS on cognitive function using RGT. We found that daily VNS, administered immediately following the training sessions of RGT, resulted in increase in the number of ‘good decision makers’.

In the last few years, large-scale neural oscillations have been acknowledged to play a primary role in fundamental cognitive function. Ample evidence suggests that neurons transmit information not only by changing their firing rates but also in terms of the timing of the spikes corresponding to the ongoing neuronal oscillations^21^. Furthermore, the induction of synaptic plasticity is favored by coordinated action potential timing across neuronal networks^22^, giving rise to oscillations of different frequencies in the local field potential (LFPs). These field potential oscillations have been shown to modulate local spike timing^23^ and in particular Rutishauser *et al*. have shown that close coordination of spike timing with the local theta frequency band oscillations predicts the formation of memory in humans^24^.

The anterior cingulated cortex (ACC) is a major cortical area of the limbic loop system, integrating emotion and cognition. It has been shown that the basolateral amygdala (BLA) and the ACC form an interconnected neural circuit that may mediate certain types of decision-making processes^25^. Accordingly, in this study a multiple-electrode array recordings technique was used in conscious rats, to characterize the VNS-induced increases in theta band power in the BLA and ACC. Moreover, the association between the locking of the ACC spikes to the phase of the theta oscillations in the BLA (cross-area spike-LFP-phase locking) following VNS was quantified.

## Results

### Effects of Electric Vagal Stimulation on Decision-Making of Rats

One control (sham EVS) rat was excluded as during the training period it failed to reach the required criterion. At the beginning of RGT training two rats failed the test to induce a behavioral response (flattening the ears, taking a fixed posture) by high intensity VNS stimulation which is a criteria of successful implantation of the cuff electrode. Four sham VNS rats and three VNS rats developed a form of spatial preference behavior for one side of the apertures during training and maintained this side-preference for the whole 60 min test. There was no statistical difference between the two groups for this type of preference behavior (VNS, 7.5% vs. control, 12.5%, Fisher’s exact test, p > 0.05). These rats were not included in the final analysis as their preferred choices in the test were not dependent on the decision-making process based on the different outcomes.

**Figure 1:**
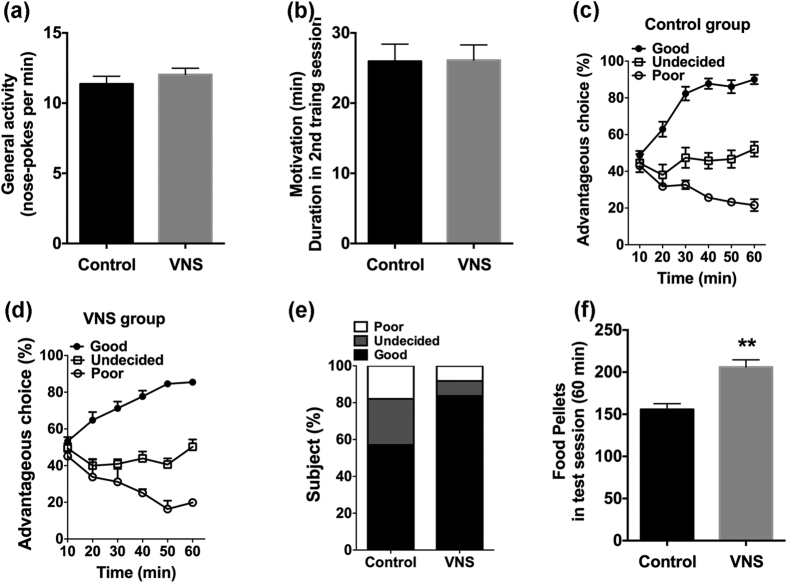
The performance of control rats and rats with VNS treatment in the rat Iowa gambling task. (**a,b**) No difference was detected in the general activity and motivation between the control and VNS rats. (**c,d**) The ratios of advantageous choices across the 60 min test identify three types of decision-makers in both control and VNS rats. (**e**) The different proportions of the three types of decision-makers in control and VNS rats. (**f**) The food pellets obtained during the test for control and VNS rats. Results are presented as mean ± SEM, n = 28 for control group and n = 37 for VNS group. Statistical significance was determined by student t-test, **p < 0.01.

The average food intake per body weight was not significantly different between the two groups (0.073 ± 0.004 in control rats vs. 0.067 ± 0.007 in VNS rats, t(63) = 0.47, p > 0.05). The average body weights were not significantly different between the two groups (313.7 ± 7.5 vs. 306.8 ± 8.6, t(63) = 0.51, p > 0.05) and no difference was detected in the general activity - number of nose-pokes per min - (VNS, 11.37 ± 0.54 vs. control, 11.66 ± 0.44; t test, t(63) = 0.93, p > 0.05, Fig. 1a) or motivation (VNS, 25.96 ± 0.46 min vs. control, 25.75 ± 0.16 min; t test, t(63) = 0.25, p > 0.05, Fig. 1b) between two groups. The performance of the control rats and rats with VNS during the RGT test are shown in Fig. 1c,d. Good decision-makers indicates those rats who chose randomly at first, then orientated their preference progressively toward more advantageous options and ended up making more than 70% advantageous choices during the last 20 min. Conversely, poor decision-makers developed a stable preference for the adverse options, making less than 30% advantageous selections during the last 20 min of the test. The number of choices of each option (A, B, C, D) of each rat are shown in Table 1 in the Supplementary Information as the percentages of advantageous choices during last 20 min.

In the VNS group, the proportion of good decision-makers increased compared with the controls (VNS, n = 31 (83.7%) vs. control, n = 16 (57.1%), Fisher’s exact test, p < 0.05). The difference in the proportions of the three types of decision-making behavior (good, bad, undecided) between the two groups was significant (Mann-Whitney U test, U = 380.00, p = 0.018, Fig. 1e). The mean food reward obtained during the RGT by the VNS rats was significantly more than that of the controls (VNS, 206.0 ± 8.57 vs. control, 152.1 ± 6.09, t test, t(63) = 4.59, p < 0.01, Fig. 1f).

### Augmented theta activity in BLA and ACC following VNS

**Figure 2:**
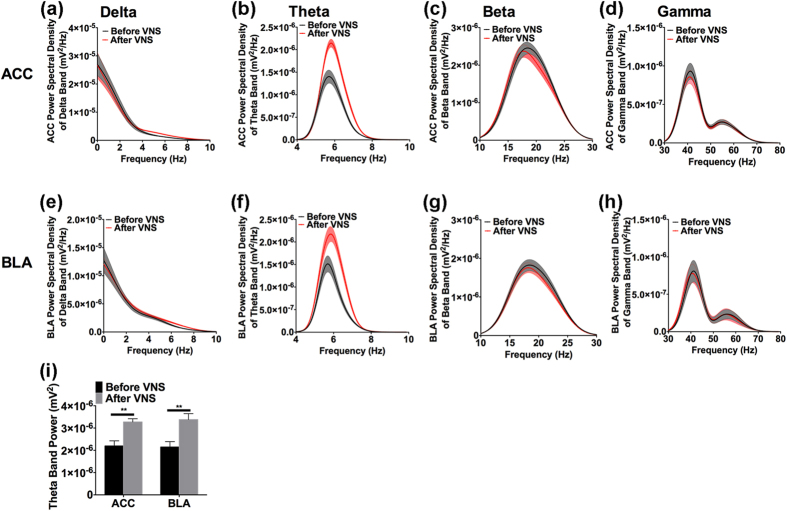
Power spectral density analysis of four frequency bands of the LFP in the BLA and ACC before (black line) and immediately after VNS (red line). (**a,e**) Distributions of power spectral density from 0 to 10 Hz showed that delta band (1–4 Hz) PSD did not obviously change following VNS in either the ACC (**a**) or the BLA (**e**). (**b,f**) Distributions of theta band (4–10 Hz) PSD increased following VNS in both the ACC and the BLA. (**c,g**) Beta band (10–30 Hz) PSD did not change following VNS in either the ACC or the BLA. (**d,h**) Gamma band (30–80 Hz) PSD did not change following VNS. The trough at 50 Hz was induced by the 50 Hz notch filter used during recording. (**i**) Area under curve showed that only the theta band power increased significantly following VNS in both the BLA and the ACC. Results are presented as mean ± SEM. n = 6. **p < 0.001.

In the 8 rats implanted with multiple-channel electrodes, two rats failed the testing for successful implantation of the cuff electrode, the remaining 6 rats all showed good-decision making behavior (Table 1 in Supplementary Information). Averages of the Fourier transform for four frequency bands of the field potentials are depicted in Fig. 2. The area under the curve (AUC) of the theta band power in the BLA and ACC are shown in Fig. 2i. Following VNS, the theta band power increased significantly from 2.14 × 10^−6^ ± 2.40 × 10^−7^ mV^2^ to 3.37 × 10^−6^ ± 2.69 × 10^−7^ mV^2^ in the BLA (t(5) = 26.5, p < 0.001), and from 2.19 × 10^−6^ ± 2.31 × 10^−7^ mV^2^ to 3.27 × 10^−6^ ± 1.45 × 10^−7^ mV^2^ in the ACC (t(5) = 6.724, p < 0.001). No significant changes in power were observed in the other frequency bands. Therefore, these data revealed that only theta band activity was influenced following VNS, suggesting that VNS affected the theta frequency band activity related to cognitive functions.

### Facilitated Spike-Field Coherence within the BLA Following VNS

**Figure 3:**
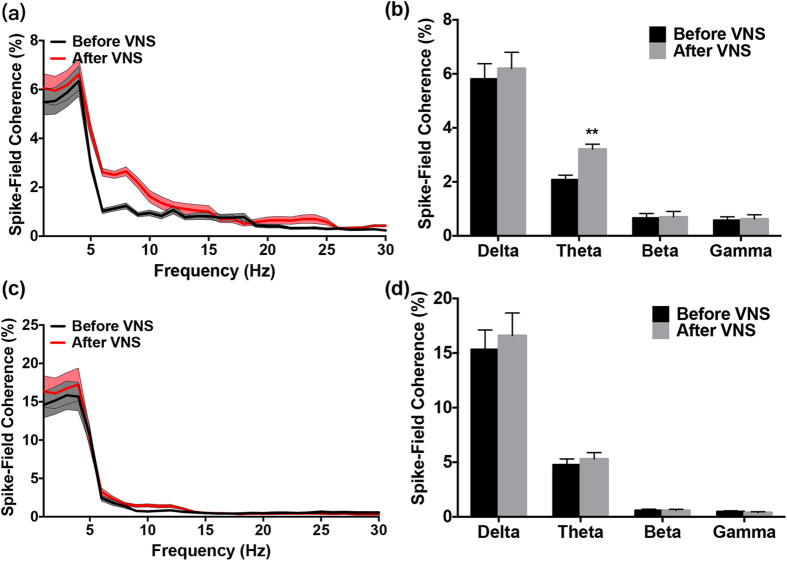
The Spike-Field coherence within the BLA increased immediately following VNS. (**a**) Distribution of the spike field coherence (SFC) spectrum within the BLA before VNS (black line) and immediately after VNS (red line). (**b**) The mean SFC values in the four frequency bands. The SFC in the theta band was increased significantly following VNS. (**c**) Distribution of the SFC within the ACC before and immediately after VNS. (**d**) No significant change was found in the mean SFC values of the four frequency bands. Results are presented as mean ± SEM, n = 71 in the BLA and n = 60 in the ACC. Statistical significance was determined by t test, **p < 0.001.

In the current study, spike-field coherence (SFC) was used to quantify the alteration in the spike timing-LFP relationship within the BLA and ACC before and after VNS. The SFC value is expressed in percentage and varies as a function of frequency. The SFC takes values from 0% to 100%, with 100% indicating all spikes follow a particular phase of oscillation in this frequency, while 0% reflects spikes firing completely at random. There were 71 neurons recorded in the BLA of 6 rats, including 5 neurons have spike(s) within 2 ms refractory period, and the mean percentage of spikes was 2.10% ± 0.32% within 2 ms (n = 5). The SFC distributions for various frequencies are presented in Fig. 3. We found a significant difference in the average SFC after VNS in the theta range (t(69) = 5.112, p < 0.001; Fig. 3b). SFC values were increased in the theta band from 2.028 ± 0.1669 before VNS to 3.214 ± 0.1804 after VNS. No significant changes were observed in the other three frequency bands. 60 neurons recorded in the ACC were used to calculate the SFC within the ACC. The mean percentage of spikes was 2.25% ± 0.23% within 2 ms (n = 4). No significant change was found in the theta band (t(58) = 0.456, p > 0.05, Fig. 3d). These results suggest that spike-field coherence in the theta band oscillations is changed in BLA after VNS.

### Increased Proportion of Neurons Phase-Locked to Theta Oscillations within the BLA Following VNS

**Figure 4:**
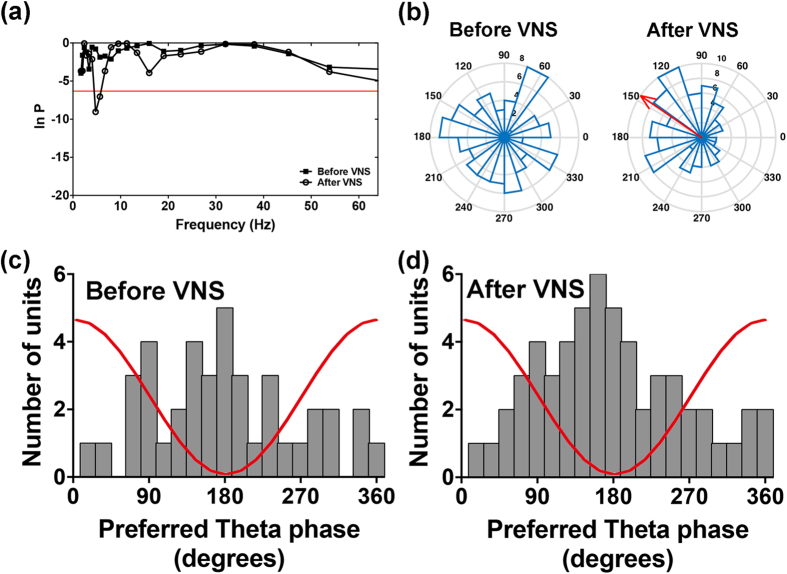
Spike phase-locking to theta oscillations within the BLA. (**a**) Test of significance of phase-locking as a function of frequency (1–64 Hz). The threshold (red line) for significant phase-locking was set to p = 0.0023 (0.05/22, Bonferroni corrected). The neuron which was un-phase-locked before VNS exhibited maximal phase-locking at 4.75 Hz after VNS. (**b**) The polar histograms of the spike-field phase distribution of the same neuron shown in (**a**). The neuronal action potential fired at random angles of the theta cycle oscillation before VNS (left panel). While the majority of spikes of this neuron fired at 150° after VNS (right panel), which is indicated by the red arrow (vector length R = 0.47). (**c,d**) Histogram of the distribution of the preferred phases of all phase-locked neurons before and after VNS, n = 40 in (**c**) and n = 56 in (**d**). The figure shows more phase-locked neurons preferred to fire close to the trough of the oscillation. The red line is a schematic of the theta cycle.

We next considered whether the phase distributions of the LFP in relation to the neuronal firing in the BLA were changed immediately following VNS. As expected, we found that 22.5% of neurons (16 of 71 neurons) fired spikes randomly to the phase of the LFP oscillations in the theta range before VNS and became phase-locked to the theta oscillations following VNS (Fig. 4a, p < 0.0023, 0.05/22, Rayleigh’s test). The frequency with the maximal phase-locking in the Rayleigh test was 4.75 Hz. Example phase distributions of a neuron un-phase-locked before VNS and phase-locked after VNS are presented as polar-histograms (Fig. 4b). The un-phase-locked neuron displayed random firing. However, this neuron became phase-locked after VNS and showed most spikes firing close to 120° of the theta cycle with a mean-phase at 150°. Before VNS, 56.3% (40 of 71 neurons) showed phase-locking close to the trough of theta oscillation (Fig. 4c). In contrast, a significantly increased proportion of neurons (78.8%, 56 of 71 neurons, t(19) = 3.559, p < 0.01) showed phase-locking to the theta range following VNS (Fig. 4d). Together, the results indicate VNS causes an increase in the concentration of the distribution of the neuronal spikes in relation to the phase of the ongoing LFPs within the BLA.

### Increased Proportion of ACC Neurons Phase-Locked to the BLA Theta Oscillation Following VNS

**Figure 5:**
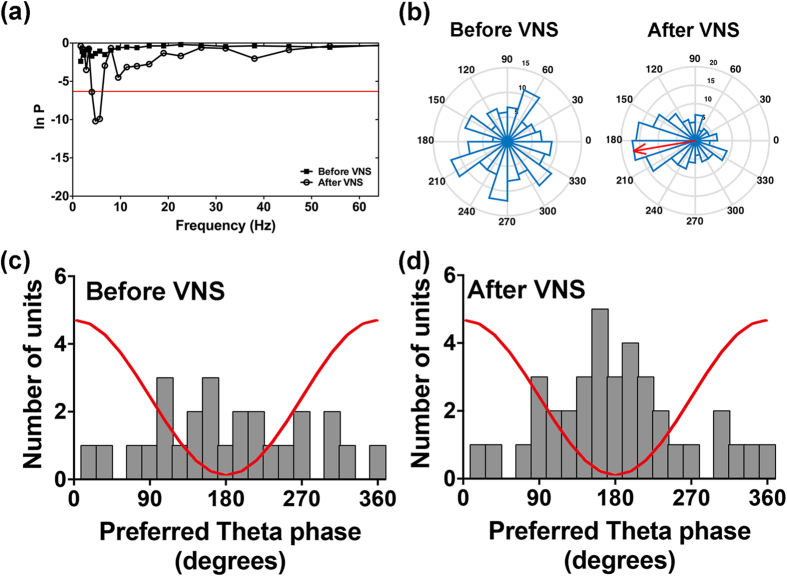
Phase-locking of the spikes of the ACC neurons to the theta oscillations in the BLA. (**a**) Test of significance of phase-locking. The neuron which was un-phase-locked before VNS exhibited maximal phase-locking at 5.65 Hz after VNS. (**b**) The polar histograms of the spike-field phase distribution of the same neuron shown in (**a**). The un-phase-locked neuron fired randomly before VNS (left panel) and became phase-locked to the theta cycle at 189° after VNS (right panel, vector length R = 0.53). (**c,d**) Histogram of the distribution of the preferred phases of all phase-locked neurons before and after VNS, n = 26 in (**c**) and n = 37 in (**d**). More phase-locked neurons preferred to fire close to the trough of the oscillation.

Phase synchronization is a fundamental neural mechanism to support neural communication and neural plasticity between regions, and may have relevance for many cognitive processes^26^. In the present study, we investigated whether the distributions of the ACC neuronal firing in relation to the phase of the local field potential of the BLA were changed following VNS. 18.3% of neurons (11 of 60 neurons) which before VNS fired spikes at phases random to the LFP oscillations in the theta range became phase-locked to the theta oscillations (Fig. 5a, p < 0.0023, 0.05/22, Rayleigh’s test). The frequency with the maximal phase-locking in the Rayleigh test was 5.65 Hz. Example phase distributions of a neuron un-phase-locked before VNS and phase-locked after VNS are presented as polar-histograms (Fig. 5b). The neuron randomly fired before VNS and fired spikes almost between 144° and 216° of the theta cycle after VNS with a mean-phase of 189°. Figure 5c revealed 43.3% (26 of 60 neurons) showed phase-locking close to the trough of theta oscillation before VNS. In contrast, a significantly increased proportion of neurons (61.7%, 37 of 60 neurons, t(19) = 2.604, p < 0.05) revealed phase-locking to the theta range following VNS (Fig. 5d). In comparison the proportion of BLA neurons phase-locked to the theta oscillations in the ACC displayed no significant changes after VNS (t(19) = 1.453, p > 0.05). Together, the results indicate VNS causes an increase in the concentration of the distribution of the neuronal spikes in the ACC in relation to the phase of the ongoing LFPs in the BLA.

### Increased Synchronization of Theta Activities in the ACC and BLA After VNS

**Figure 6:**
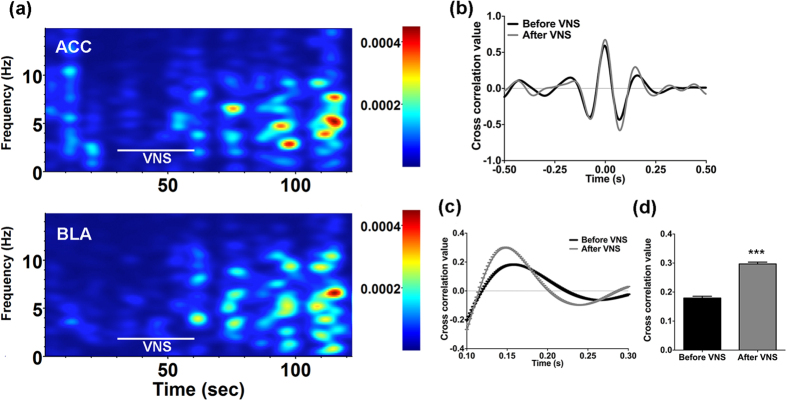
The synchronization of theta activities between the ACC and the BLA increased following VNS. (**a**) Two minutes colored power spectra of LFPs in the ACC and the BLA show synchronized theta oscillations after VNS. (**b**) The averaged cross-correlograms before and after VNS. (**c**) The second positive peaks of the cross-correlograms which are shown in (**b**), which corresponds to the theta band activity. (**d**) Histograms showing the averaged cross correlation value before and after VNS during spontaneous activity. Theta band correlation increased following VNS. Results are presented as mean ± SEM, n = 6. Statistical significance was determined by student t-test, ***p < 0.0001.

**Figure 7:**
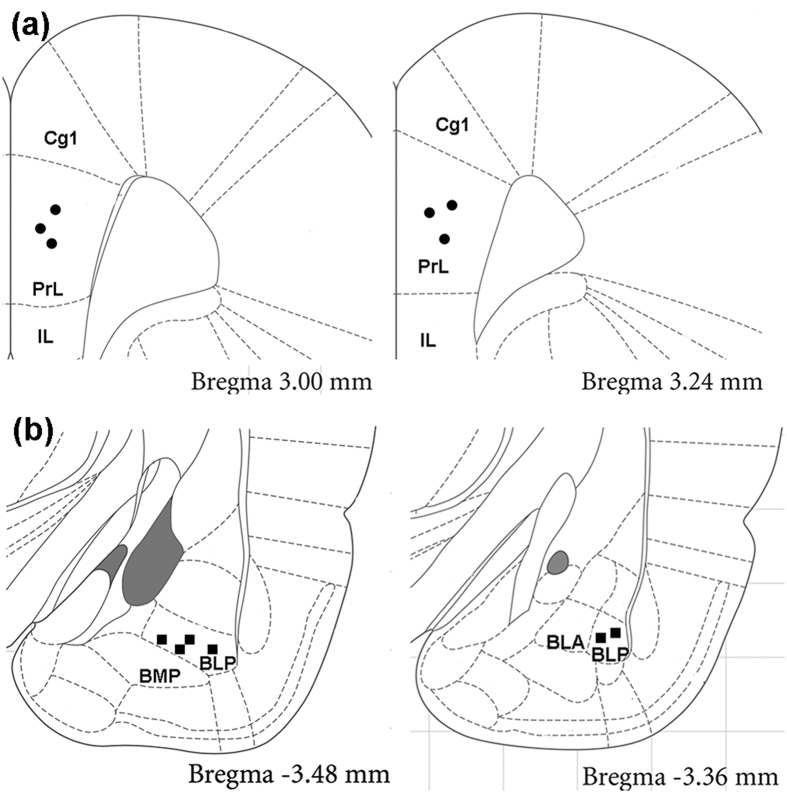
Recording sites in the ACC (**a**, solid circles) and the BLA (**b**, solid squares) in rats. The drawing is adapted from our previous publications^15^,^37^ according to the present study. Cg1, cingulate cortex, area 1; PrL, prelimbic cortex; IL, infralimbic cortex; BLA, basolateral amygdaloid nucleus, anterior; BLP, basolateral amygdaloid nucleus, posterior; BMP, basomedial amygdaloid nucleus, posterior.

To further examine the functional connectivity between the ACC and BLA, we compared the LFP during 30 s spontaneous periods from before and after VNS in rats. Time-varying power spectra analysis revealed that the dispersed neuronal activities in the ACC and BLA became concentrated in the theta band following VNS (Fig. 6a). Cross-correlation analysis allows quantitative evaluation of the synchronization of activities in the LFP. The second positive peak represents the synchronized activities of the LFP in the theta band as this corresponds to a lag time of from 0.1 to 0.25 s corresponding to the theta band in 4–10 Hz (Fig. 6b). By averaging the cross-correlograms and taking the second positive peak as a quantitative measure (Fig. 6c), a significant difference was detected (t(10) = 13.31, p < 0.0001; Fig. 6d). The correlation value for spontaneous conditions increased from 0.179 ± 0.006 before VNS to 0.299 ± 0.006 after VNS in rats (n = 6). The result suggests increased synchronization at rest between the BLA and ACC. This indicates that the BLA and ACC may closely interact for dynamic information transfer after VNS. Finally, at the end of electrophysiological recording, the recording sites were checked by Nissl staining, electrode placements in the ACC are shown in Fig. 7a, and in the BLA are shown in Fig. 7b. Examples of Nissl-stained sections with visible marking lesions are also showed in Supplementary Fig. 2.

## Discussion

Decision-making is the result of the integration of several brain areas and has emerged as a crucial theme in neurophysiological studies of cognition. Rodents are individuals that can exhibit human-like cognitive characteristics, such as the ability to learn and reason using causal knowledge^27^. In this study we performed the RGT to investigate the effects of VNS on cognitive function in normal subjects. Three types of decision-making behaviors, i.e. good, undecided, and poor decision-makers, could be identified^14^,^28^ in both the control rats and the rats with VNS. The current study applied electrical stimulation of the left cervical vagus nerve (400 μA, 1 Hz, 30 s) in rats immediately following the RGT training phase. This procedure led to an increase in the proportion of good decision-makers, and a decrease in the proportion of maladaptive decision-makers compared with using sham VNS as a control. The stimuli parameters are comparable with previously reported ones, as having a decision making-enhancing effect. Similar paradigms of VNS have been applied in humans and rats and have shown enhanced working memory in humans^13^. We determined that VNS had no general effect on the motivation to actively retrieve food rewards (duration of the last training session before reaching the criterion). Furthermore, no difference was detected in the general activity (number of nose-pokes in the four holes in the last training phase). These observations suggested that VNS did not directly affect the overall ability of rats to complete the RGT.

In studies of lesion or psychiatric patients research has uncovered the specific prefrontal cortical (PFC) areas that mediate decision-making, notably the orbitofrontal cortex, the prefrontal cortex, and the anterior part of the cingulate cortex^14^,^16^,^17^,^18^. It has been demonstrated that the ACC plays a crucial role in selecting proper actions when faced with different benefits in an uncertain environment^29^ by signaling error-likelihood^30^. The ACC also plays a key role in choosing appropriate actions when the environment is dynamic^31^. Extensive functional and neuroanatomical evidence supports the concept that the ACC has functionally distinct subregions. Vogt^32^ defines the midcingulate region as the ventral division of the ACC, signified as area 24′ in the primate. The division of the ACC into midcingulate and rostral cingulate is determined by the different cytoarchitecture and connections^32^,^33^,^34^. From a connection point of view, rostral area 24′ in the rat receives most mediodorsal thalamic^35^ and amygdala^36^ afferents. In the current study we recorded the neuronal activity of the ACC in the rat, which is a major cortical area of the limbic system, integrating emotion and cognition. As we have previously described^37^,^38^,^39^ the ACC is defined in this case as the anterior cingulate cortex, area 2 (Cg2), and the prelimbic cortex with overlying cingulate cortex, area 1 (Cg1)^40^.

In addition to prefrontal cortical areas, several studies in the literature highlight the role of an intact amygdala in advantageous decision-making in the gambling task^12^,^41^. It has been shown that patients with highly localized amygdala lesions performed worse on the IGT and were impaired in judging emotional facial expressions^12^,^18^. These patients also failed to show risk seeking for losses^42^. Animal studies have shown that BLA lesions induced risky choice in a RGT^18^ comparable to the effects of amygdala damage in humans^12^, indicating that this region makes similar contributions to decision-making across species. There is anatomical and functional evidence for direct, ipsilateral projections from BLA to mPFC^43^,^44^ suggesting a tight functional interaction.

Theta band brain oscillations have been acknowledged to play a role in extensive cognitive functions^45^. Theta band activity of LFPs is mainly observed in the limbic system including the hippocampus and the cingulated cortex as well as in the PFC^46^. A human study has demonstrated that theta-frequency phase-locking of single neurons played an important role in memory strength^24^. In this study, increases in coherence between spikes and theta oscillations in the BLA neurons in the rats, immediately following moderate electrical VNS, were clearly demonstrated. The SFC is independent of the LFP power spectrum and the number of spikes, and is therefore immune to changes in these parameters. This allowed us to distinguish between changes in spike-field synchronization and changes in the regularity of oscillatory patterns that are reflected in enhancement of the spectral power of the field. In addition, to advance the understanding of the timing relationship between spikes and ongoing theta oscillation, it is critical to investigate the angular distributions of spikes with the theta oscillations. This information then allows the significance of phase-locking of spikes in theta oscillations to be clarified^24^. We found that 56.3% of BLA neurons fired spikes that were phase-locked to the LFP oscillations in the theta range before VNS. The frequency with the maximal phase-locking in the Rayleigh test was 4.75 Hz, with maximal activity during the descending phase and at the trough of the oscillations. In contrast, 78.8% of BLA neurons showed phase-locking at the theta range following VNS suggesting that VNS facilitates coordinated phase distribution of BLA neuron spikes in the theta oscillations of the LFP.

It is clear that communication between brain areas involves phase synchronization of oscillations^47^,^48^. The phase of the oscillation regulates exactly when gatherings of neurons spike, thus two brain areas with increased phase synchrony will have improved synaptic interaction and information exchanges^26^. Previously, the reciprocal connections between the BLA and medial PFC, including the ACC, have been clearly exhibited. Indeed, an interconnected neural circuitry between the BLA and the ACC region of the medial PFC guides behaviors in certain types of cost–benefit decision-making task^25^. In the current study cross-correlation and time-varying power spectral analysis of the theta oscillations revealed a pattern of dispersion of theta band activity during the basal period, and increases in correlation values immediately following VNS. Moreover, the increased LFP-synchronization between the BLA and the ACC was also associated with greater locking of ACC spikes to the phase of the theta oscillations in the BLA. The cross-area spike-LFP-phase locking following VNS suggested that reciprocal connections between the BLA and the prefrontal cortex provide a critical circuit for using incentive information to guide behavior. For example, humans with damage to either amygdala or prefrontal cortex are impaired in the capacity to assess and use the value of predicted outcomes to guide their actions in the Iowa gambling task^12^. Similar observations were obtained in monkeys^49^. Our findings support a role for input from the BLA to the ACC in facilitating the encoding of information about expected outcomes in the ACC. Our results from the RGT and multiple electrophysiological recordings are consistent with published observations that bilateral inactivation of the BLA impaired decision-making; further a unilateral BLA inactivation combined with a contralateral ACC inactivation also impaired decision-making^25^. It appears that the sequential transfer of information via corticipetal BLA/ACC connections may guide response variety when assessing the value of an anticipated outcome relative to the costs of a particular action.

It would be important in further studies to clarify what physiological stimuli activate the vagal afferents that modulate decision-making. For instance, Cholecystokinin-octapeptide (CCK-8), which is a gastrointestinal hormone released during feeding^50^,^51^, acts on vagal afferent fibers. Our previous electrophysiological studies in rats have demonstrated that CCK stimulates vagal afferent fibers^52^,^53^ to modulate various gastrointestinal functions. Flood *et al*., have shown that administration of CCK-8 acts on vagal afferents to enhance memory retention in the mice after aversive training^54^. Further studies are needed to determine if CCK enables or modulates cognitive function, such as decision-making, by acting on vagal afferent fibers.

In this study VNS was applied after training session. Both other investigators^9^ and ourselves, have shown that post-training VNS facilitate memory consolidation^11^. Given this line of evidence, it may be argued that the decision-making improvement in this study can be explained by the mechanism of memory improvement produced by VNS. Indeed, decision-making is a process that is partially dependent on memory systems^55^, and impaired working memory can compromise decision-making and performance on the gambling task. Therefore, it is possible that VNS exerts memory improvement, which in turn helps improve decision-making. Obviously, these potential mechanisms are speculative at this early stage, but they raise intriguing questions for future investigations.

Note that the therapeutic effects of VNS often enhances over months, or longer, in some patients^56^. Therefore, VNS-induced brain plasticity may vary between acute and chronic states of VNS. Thus, the immediate effects following VNS reported here may not reflect the longer-term effects of the VNS as a therapy. In addition, when applied with different use parameters, VNS may be selectively “targeted” to modify different brain regions, with attendant “focusing” of behavioral effects. Furthermore, VNS has been shown to activate both noradrenergic and serotonergic cell body areas^57^,^58^. Therefore, in this study electrophysiological effects of VNS measured after the training session cannot be directly linked with the decision making behavioral effects measured in the RGT probe tests. It is necessary to combine electrophysiological recordings of functional neuronal activities with VNS with respect to the use parameters in behavioral animal experiments.

In summary, in the behavioral animal, moderate electrical vagus nerve stimulation following training sessions facilitates decision making in rats. In parallel, we observed greater phase locking of the theta oscillations in the BLA to the ACC spikes immediately following VNS. Our findings suggest that theta synchrony may reveal more proficient trial and receive during gambling task training, and might also help endorse information into memory. These data are consistent with the theory that oscillatory synchrony, and particularly phase synchronization, is an overall principle for integrating information between different cortical and subcortical areas during complex cognitive processing. The data may serve as a basis to develop fundamental notions regarding neurophysiologic biomarkers for therapeutic VNS in cognitive impairment.

## Methods and Materials

### Animals

Experiments were performed on adult male Sprague-Dawley rats (300–350 g). All experimental procedures were conducted according to the guidelines laid down by the NIH in the US regarding the care and use of animals for experimental procedures and were approved by the committee on the Use and Care of Animals at the City University of Hong Kong and the licensing authority for conduction experiments of Department of Health of Hong Kong (No. 15–80 in DH/HA&P/8/2/5).

### Implantation of Vagus Nerve Cuff Electrode and Implantation of Recording Electrodes

The bipolar cuff electrode system was made as described previously^8^. The VNS cuff electrode implantation procedure was adapted from our prior studies^11^. Two 16-channel micro-wire electrode arrays (4 × 4) were inserted into the prelimbic area of the ACC and the ipsilateral BLA^15^,^37^,^40^. Details of procedures can be found in the Supplementary Information.

### Rat Gambling Task

The RGT has been developed to test the decision-making capacities of rats via a conflict between immediate and long-term gratification (food reward). During the training stage, rats gradually learned the association between the nose-poke action and the release of a food pellet. Electrical vagal stimulation was given to rats immediately after training^10^. Rats were free to make choices between four apertures (A–D), however, different choices were associated with different outcomes. The percentage of advantageous choices ((C + D)/(A + B + C + D) × 100%) made during the last 20 min and the food rewards obtained across the test were used to identify the decision-making behavior of rats. The details of the apparatus and experimental procedures for gambling task have been described in prior studies^15^,^28^,^59^, and can be found in the Supplementary Information.

### Electrophysiology of Multiple-Channel Recording with Vagus Nerve Stimulation

Following RGT training, the rat was returned to its home cage. Electrophysiological recordings were performed in the home cage during quiet awake conditions on each day. Recordings lasted for a total of 120 seconds. The basal firing rate and LFP were assessed over 30 s to quantify the resting discharge and LFP power. Vagal nerve stimulation (VNS 400 μA, 0.5 ms pulse width; 1 Hz; 30 s duration) was performed from 30 to 60 s to stimulate the vagal C-fiber. Recordings were continued for 60 seconds following cessation of VNS. Spikes and LFPs were analyzed using data 0–30 s, and 60–90 s. LFPs were band-pass filtered (0.05–200 Hz with 50 Hz notch filter, 4-pole Bessel) and sampled at 1 kHz. Spikes were filtered (0.3–5 kHz, 4-pole Bessel) and sampled at 40 kHz.

### Histological Identification of Recording Sites

At the end of the recordings, a small electrode lesion (anodal DC current, 50 μA for 30 s) was made as we have previously described^15^,^28^.

### Multiple-Channel Neural Data Analyses

#### Spectral analysis

The power spectral densities (PSDs) in the delta (1–4 Hz), theta (4–10 Hz), beta (10–30 Hz) and gamma (30–80 Hz) frequency bands were computed. In order to achieve this, the raw LFPs were filtered between 1 and 100 Hz using non-causal zero-phase-shift filter (fourth-order Butterworth). Then the PSDs were calculated by multi-taper estimates with seven tapers, 2^13^ frequency bins in the range [0, 500 Hz] (NeuroExplorer 5, Plexon, Dallas, TX) and 50% overlapping windows (window durations were 2^14^ data points). The PSD curve was smoothed with a Gaussian filter (15 bins running average). The band power was defined as the AUC of the corresponding frequency domain. The PSD values from each animal were averaged over the 16 channels in the ACC and BLA separately. The spectrum units were normalized by raw PSD, so that the sum of all the spectrum values equals to the mean squared value of the signal.

#### Spike Sorting

The single unit spike sorting was conducted using Offline Sorter Version3 software (Plexon Inc.). Spikes were identified when a minimum waveform amplitude threshold of 4 SDs higher than the noise amplitude was reached. All waveforms in each channel were automatically isolated as distinct clusters by principal component analysis (PCA). Manual checking was performed to ensure consistent spike waveforms and separate cluster boundaries. A single unit was defined using the criterion of finding <3% of the spikes in the refractory period of 2 ms^60^ (Fig. 1 in Supplementary Information).

#### Computing the Spike-Field Coherence within BLA Regions

SFC was used to measure the phase synchronization between action potentials and field potential oscillations^24^. To compute the SFC within the BLA or ACC, the spikes and LFP recorded from the same channel were used in the analyses. For every spike, a segment of the LFP data centered on the spike ±480 ms were averaged to calculated the spike-triggered average (STA). Then the frequency spectrum of the STA (fSTA) was calculated using multitaper analysis, which uses a series of discrete prolate spheroidal sequences (7 tapers) to give estimates of the PSD. The average of the spectra in each frequency results in the spike triggered power (STP(f)). Finally, the SFC was calculated as the fSTA over STP(f) as a percentage: SFC(f) = [fSTA(f)/STP(f)] × 100%.

#### Phase-Locking of Single Neurons to the Theta Oscillation

To further check the phase-locking of a single to the LFP oscillations within the BLA and the exact phase of the phase-locking, the Rayleigh test was used to compare against uniformity. 22 frequencies (from 1.6 to 64 Hz) were selected as f = 2^x^ with x = [6/8, 8/8, 10/8, …, 48/8] and p values (0.0023 = 0.05/22) calculated for each. To analyze the phase-locking within the BLA, the spikes and LFP recorded from the same channel were used in the analyses. To ensure the validity of the statistical results, neurons that had at least 50 spikes were used for Rayleigh test. The LFP was convolved with a series of Morlet wavelets centered at each of the specified frequencies and with a length of four cycles. The Morlet wavelet-transform results in a matrix of vectors, which represent the amplitude by length, and the phase by angle. The circular mean of the spike phases was calculated by taking the weighted sum of the cosine and sine of the angles, finally resulting in, the mean angle and mean vector length (R) over the number of spikes^24^.

#### Synchronized Theta Oscillations between BLA and ACC

For the LFPs, cross-correlation analysis was not affected by changes in the amplitude, but was sensitive only to changes in phase between the two regions. Our interest was focused on the time coupling between the BLA and ACC, hence we used cross-correlograms (Neuroexplorer 5) to evaluate the synchronization of the theta band activities between BLA and ACC. LFPs from the BLA and ACC were averaged over 16 channels separately before aligning, and the LFP in the BLA was chosen as the reference. Pearson correlation values were calculated with a lagging time from −0.5 to 0.5 s with 2 ms bins. The cross correlation curves were smoothed by a Gaussian filter (5 bins running average). The second positive peak was chosen as a quantitative measure because its location at 0.2 s lagging time represents theta activity at about 5 Hz.

## Additional Information

**How to cite this article**: Cao, B. *et al*. Vagus Nerve Stimulation Alters Phase Synchrony of the Anterior Cingulate Cortex and Facilitates Decision Making in Rats. *Sci. Rep.* **6**, 35135; doi: 10.1038/srep35135 (2016).

## Supplementary Material

Supplementary Information
