# Supplementary material for: Vagus Nerve Stimulation Alters Phase Synchrony of the Anterior Cingulate Cortex and Facilitates Decision Making in Rats
Source: Sci Rep. 2016 Oct 12;6:35135. doi: 10.1038/srep35135 (PMC5059720; doi:10.1038/srep35135)
Supplement: Supplementary Information [file srep35135-s1.pdf]

Supplementary Information

Vagus Nerve Stimulation Alters Phase Synchrony of the Anterior Cingulate Cortex and Facilitates Decision Making in Rats

Bing Cao, Jun Wang, Mahadi Shahed, Beth Jelfs, Rosa H. M. Chan and Ying Li*

Materials and Methods

Animals

Experiments were performed on adult male Sprague-Dawley rats (300–350 g). A total of seventy-five rats were used. The animals were housed one per cage and settled in a temperature controlled room (25℃) with 12h light/dark cycle. They were habituated for at least 5 days before any experiment and given access to food and water ad libitum except for the period of the rat gambling task (RGT). All experimental procedures were conducted according to the guidelines laid down by the NIH in the US regarding the care and use of animals for experimental procedures and approved by the committee on the Use and Care of Animals at the City University of Hong Kong and the licensing authority for conduction experiments of Department of Health of Hong Kong (No.15-80 in DH/HA&P/8/2/5).

Cuff Electrode System

The bipolar cuff electrode system was made as described previously[1,2]. Briefly, Teflon coated silver wires were inserted in parallel into PVC tubing without insulation and with a 1.5 mm separation. This PVC tubing served as a cuff of electrodes and was used to give electrical VNS to the rats. To decrease the activation of vagal efferent, the anode of the electrode was configured as the distal lead and the

cathode as the proximal lead which made direct afferent action potential propagation preferential by creating an anodal block at the distal lead.

Implantation of Vagus Nerve Cuff Electrode and Multiple-Channel Recording Electrodes

Rats were anesthetized with pentobarbital (i.p. 50 mg/kg). Body temperature was maintained at 37℃ with a temperature-controlled heating pad. Depth of anesthesia was determined to be enough by frequently observing the absence of heart rate alterations, and in a separate set of experiments, the withdrawal response after pinching the skin without immobilization. The implantation procedure used here was adapted from prior studies[2]. Briefly, sagittal incision was made above the clavicle. The left omohyoid and sternomastoid muscles were separated to isolate the nerve and insert the electrode. The electrode leads were drawn between the skin and subcutaneous fasciae to the dorsal aspect of the neck and exited through a 0.5-cm incision. Following implantation surgery, as a criterion for good contact between electrode and nerve, the vagus nerve was stimulated at 0.2 mA which proved sufficient to stop respiration in anesthetized animals.

Following implantation of the VNS cuff, 8 of 42 rats with the cuff electrode were chosen randomly to implant multiple-channel recording electrodes in the BLA and ACC. The skull was exposed and two small holes (1-2 mm wide) were drilled above the right side for recording electrodes implantation. Four stainless bone screws were inserted into the skull surrounding the surgical openings and the dura mater was subsequently removed. Because the almost exclusively caudal regions of the BLA ascend projections to the ACC unilaterally[3], two 16-channel micro-wire electrode arrays (4×4) were inserted into the prelimbic area of the ACC (anterior-posterior (AP)

= 3.0-3.3, Medial-lateral (ML) = 0.6-1.0, dorsal-ventral (DV) = 2.8-3.5 mm from dura) and the ipsilateral BLA (AP -3.3 to -3.6, ML 5.0-5.3, depth 6.5-7.5 mm). This array consisted of 16 polyimide-insulated platinum/iridium microwires (Clunbury Scientific) and was sterilized before insertion. The microwires were arranged in 4 rows with 4 wires in each row (electrode diameter=25 μm; electrode spacing=250 μm; row spacing=250 μm; impedance=20–50 kΩ, measured with microelectrode meter Ωmega-Tip Z, WPI instruments). Finally, a silver wire from each array was wrapped around one of the bone mounting screws for grounding. Then, the recording electrodes were advanced slowly into the brain using a micropositioner until the clear neuronal firings in most recording channels were observed on-line (OmniPlex system, Plexon, USA). A mixture of mineral oil and bone wax was packed around the electrode penetration zone. Electrode arrays were secured to the rat's skull using dental cement. After the surgery, rats were injected with buprenorphine (0.1 mg/kg) as an analgesic, and 1.0 ml sterile saline for hydration, then placed on a warm heating pad for recovery.

Five days after the surgery a high intensity stimulus (1 mA, 0.5 ms pulse width, 30 Hz and 1s duration) was given through the cuff electrode to evoke a behavioral response (flattening the ears, taking a fixed posture and sometimes vocalizing), as a criterion for successful implantation in the treated rats[1,13]. The implantation of the cuff electrode was checked at the beginning and at the end of the behavioral study in each rat. The rats were allowed to adapt to the environments of the behavioral study and were handled for 3-5 min daily for 2-3 days.

Rat Gambling Task

The RGT has been developed to test the decision-making capacities in rats via a conflict between immediate and long-term gratification (food reward)[4]. The apparatus and experimental procedures for gambling task have been described in prior studies[5,6]. In short, the operant chambers (28 × 30 × 34 cm, Imetronic, Pessac, France) consisted of four apertures on the front and one food tray on the opposite wall connected to a dispenser for releasing food pellets as rewards (45 mg, TestDiet, USA). An infrared detector was equipped in each aperture and the food tray. To complete this food-driven task, before the training stage, daily food was moderately restricted for 3 days following a one-day fast such that each rat to maintained its weight at 90% of its free feeding weight.

During the training stage, each rat was placed in the chamber for 40 min each day. Rats gradually learned the association between the nose-poke action and the release of a food pellet. In order to guarantee that the selection of the nose-poke was a deliberate choice, the rats were initially trained to associate a single nose-poke with one food pellet delivery with a criterion of having obtained at least 100 pellets within a 30-min session. This was followed by two consecutive nose-pokes resulting in one food pellet delivery with the same criterion. Next, two 5 min sessions were conducted, to habituate rats for the variation in the number of pellets during the test. The first session was set so as two pellets were released after a choice was made and the second session was set to one pellet. Electrical vagal stimulation (400 μA, 0.5 ms pulse width; 1 Hz; 30 s duration) was given to rats immediately after training[2, 7, 13]. We[2], and other investigators, have observed that high intensity stimulus (1 mA) VNS evokes a behavioral response (flattening the ears, taking a fixed posture), while, no significant changes of locomotion were found during vagal nerve stimulation at 400 μA. However, Clark et al.[13] have shown that VNS (0.4 mA) given immediately after

training enhanced retention performance on an inhibitory-avoidance task in rats. In human patients, VNS at an effectively similar intensity in rodents enhanced retention of verbal learning performance[14]. Before use, the stimulation, which was delivered by isolated pulse stimulator (A-M system 2100), needed to be calibrated by oscilloscope. Rats who received sham surgery and did not receive any electrical stimulation were considered as controls. The training phase usually lasted 6-8 days.

The test procedure was performed the following day and lasted 60 min or was cut off by having obtained 250 pellets. Rats were free to make choices between the four apertures (A–D) as they were during the training phase; however, different choices were associated with different outcomes: choice A or B related to two pellets being obtained each time as an immediate reward, but had 50% probability to trigger a 222 s penalty time-out and 25% probability for a 444 s time-out , during which no pellets could be obtained; choices C or D were associated with smaller immediate rewards (one pellet each time), but also smaller penalties (25% chance for 12 s time-out, or 50% chance for 6 s time-out). During the punishment, the selected aperture remained illuminated to facilitate the association between this selection and its outcomes. Although the immediate rewards of choices A and B were twice those of C and D, in the long run the theoretical maximum benefit of C and D was five times higher than A and B. Hence, the choices C and D were more advantageous for the rats to obtain as many food pellets as possible. The number of nose-pokes per min during the last training session and the duration of the last training session were used to assess the general activity and motivation of the rats to perform the task. The percentage of advantageous choices ((C + D)/(A + B + C + D)×100%) during the last 20 min and the total food rewards obtained across the test were used to identify the decision-making behavior of the rats.

Electrophysiological signal acquisition procedure

While the rats were in the quiet awake state in their home cages, two 16-channel headstages (gain ×20) were connected to the implanted electrodes in the ACC and BLA. Both the local field potentials (LFPs) and extracellular single unit activity were recorded simultaneously using an Omniplex system (Plexon Inc.). Wideband signals were amplified (gain ×1000), high-pass filtered from 0.05 Hz, and sampled at 40 kHz. LFPs were amplified (×1000), band-pass filtered (0.05-200 Hz, 4-pole Bessel), and sampled at 1 kHz. Spike signals were amplified (×1000), band-pass filtered (300 Hz to 5 kHz, 4-pole Bessel) and sampled at 40 kHz. The recorded signals were monitored online and stored in a computer for offline analysis.

Multiple-Channel Neural Data Analyses

The detailed procedures of the multiple-channel neural data analyses were adapted from our prior studies[5,6].

Spectral analysis

To identify alterations in the theta band power spectra following VNS, we characterized the large scale brain oscillations in the BLA and ACC regions. The power spectral densities (PSD) in the delta (1-4 Hz), theta (4-10 Hz), beta (10-30 Hz) and gamma (30-80 Hz) frequency bands were computed. In order to achieve this, the raw LFPs were filtered between 1 and 100 Hz using non-causal zero-phase-shift filter (fourth-order Butterworth). Then the power spectral densities (PSD) were calculated by multi-taper estimates with seven tapers, 2^{13} frequency bins in the range [0, 500 Hz] (NeuroExplorer 5, Plexon, Dallas, TX) with 50% overlapping windows (window

durations were 2^{14} data points). The PSD curve was smoothed with a Gaussian filter (15 bins running average). The band power was defined as the area under the curve of the corresponding frequency domain. 30 s segments of LFP data from before and immediately after VNS were used to calculate the PSD, and the PSD values from each animal were averaged over the 16 channels in the ACC and BLA separately. The spectrum units were normalized by raw PSD, so that the sum of all the spectrum values equals the mean squared value of the signal.

Spike Sorting

To summarize, the single unit spike sorting was conducted using Offline Sorter Version3 software (Plexon Inc.). Spikes were identified when a minimum waveform reached an amplitude threshold of 4 SDs higher than the noise amplitude. All waveforms in each channel were automatically isolated as distinct clusters by principal component analysis (PCA). Manual checking was performed to ensure consistent spike waveforms and separate cluster boundaries. A single unit was defined using the criterion of finding < 3% of the spikes in the refractory period of 2 ms[15].

Computing the Spike-Field Coherence within BLA Regions

Spike-field coherence (SFC) was used to measure the phase synchronization between action potentials and field potential oscillations[8]. To compute the SFC within BLA or ACC, the spikes and LFP recorded from the same channel were used in the analyses. For every spike, a segment of the LFP data centered on the spike ± 480 ms were averaged to calculated the spike-triggered average (STA). Then the frequency spectrum of the STA (fSTA) was calculated using multitaper analysis, which uses a series of discrete prolate spheroidal sequences (7 tapers) to give estimates of the PSD.

The average of the spectra in each frequency results in the spike triggered power (STP(f)). As the SFC is influenced by the number of spikes, we first set the minimum number of spikes to 50 and then equalized the spikes in compared groups by randomly selecting a subsample of spikes from a larger group. Thus, the number of spikes used to calculate the single-unit SFC is unique between the comparison (before and after VNS), but varies between different single units. Finally, the SFC was calculated as the fSTA over STP(f) as a percentage: SFC(f) = [fSTA(f)/STP(f)]×100%[9]. The SFC ranges from 0 to 100%, with 0 representing a complete lack of synchronization and 100% representing perfect phase synchronization[10].

Phase-Locking of Single Neurons to the Theta Oscillation

To further check the phase-locking of a single neuron to the LFP oscillations within the BLA, and the exact phase of the phase-locking, the Rayleigh test was used to compare against uniformity. 22 frequencies (from 1.6 to 64 Hz) were selected as $f = 2^x$ with $x = [6/8, 8/8, 10/8, \ldots, 48/8]$ and a p value (0.0023=0.05/22) for each calculated[8]. To analyze the phase-locking within the BLA, the spikes and LFP recorded from the same channel were used in the analyses. To ensure the validity of the statistical results, neurons which had at least 50 spikes were used for Rayleigh test. The LFP was convolved with a series of Morlet wavelets centered at each frequency and with a length of four cycles. The Morlet wavelet-transform results in a matrix of vectors, which represents the amplitude by length, and the phase by angle. The circular mean of the spike phases was calculated by taking the weighted sum of the cosine and sine of the angles, finally, resulting in the mean angle and mean vector length (R) over the number of spikes.

Synchronized Theta Oscillations between BLA and ACC

For the LFPs, cross-correlation analysis was not affected by changes in the amplitude, but was sensitive only to changes in phase between the two regions. Our interest was focused on the time coupling between the BLA and ACC[11], hence we used cross-correlograms (Neuroexplorer 5) to evaluate the synchronization of theta activities between the BLA and the ACC. LFPs from the BLA and ACC were averaged over 16 channels separately before aligning, and the LFP in the BLA was chosen as the reference. Pearson correlation values were calculated with a lagging time from -0.5 to 0.5 s with 2 ms bins. The cross correlation curves were smoothed by a Gaussian filter (5 bins running average). The second positive peak was chosen as a quantitative measure because its location at 0.2 s lagging time represents theta activity at about 5 Hz[12].

Statistical Analysis

Results were expressed as mean ± SEM. The electrophysiological data were processed off-line using NeuroExplorer 5 (Plexon, Inc.). Statistical comparisons were performed in SPSS v13.0 (SPSS, Chicago, IL, USA). Data with equal variance were analyzed using Student's t-test. For the data with unequal variance, Mann-Whitney U test was performed alternatively. Fisher's exact test was used to compare the proportions of the subgroups of decision-makers between two groups. A value of $p < 0.05$ was considered statistically significant for all statistical comparisons.

References

1. Clark, K. B. *et al.* Post-training electrical stimulation of vagal afferents with concomitant vagal efferent inactivation enhances memory storage processes in the rat. *Neurobiol Learn Mem* **70**, 364-373 (1998).
2. Zhang, X. *et al.* Vagus nerve stimulation modulates visceral pain-related affective memory. *Behav Brain Res* **236**, 8-15 (2013).
3. Floresco, S. B. & Ghods-Sharifi, S. Amygdala-prefrontal cortical circuitry regulates effort-based decision making. *Cereb Cortex* **17**, 251-260 (2007).
4. Rivalan, M., Coutureau, E., Fitoussi, A. & Dellu-Hagedorn, F. Inter-individual decision-making differences in the effects of cingulate, orbitofrontal, and prelimbic cortex lesions in a rat gambling task. *Front Behav Neurosci* **5**, 22 (2011).
5. Mu, L. et al. Impairment of cognitive function by chemotherapy: association with the disruption of phase-locking and synchronization in anterior cingulate cortex. Mol Brain 8, 32 (2015).
6. Xu, X., Cao, B., Wang, J., Yu, T. & Li, Y. Decision-making deficits associated with disrupted synchronization between basolateral amygdala and anterior cingulate cortex in rats after tooth loss. Prog Neuropsychopharmacol Biol Psychiatry 60, 26-35 (2015).
7. Chen, S. L. *et al.* Subdiaphragmatic vagal afferent nerves modulate visceral pain. *Am J Physiol-Gastr L* **294**, G1441-1449 (2008).
8. Rutishauser, U., Ross, I. B., Mamelak, A. N. & Schuman, E. M. Human memory strength is predicted by theta-frequency phase-locking of single neurons. *Nature* **464**, 903-907 (2010).
9. Wang, J. *et al.* Theta-frequency phase-locking of single anterior cingulate cortex neurons and synchronization with the medial thalamus are modulated by visceral noxious stimulation in rats. *Neuroscience* **298**, 200-210 (2015).

10. Fries, P. A mechanism for cognitive dynamics: neuronal communication through neuronal coherence. *Trends in cognitive sciences* **9**, 474-480 (2005).

11. Guevara, M. A. & Corsi-Cabrera, M. EEG coherence or EEG correlation? *Int J Psychophysiol* **23**, 145-153 (1996).

12. Seidenbecher, T., Laxmi, T. R., Stork, O. & Pape, H. C. Amygdalar and hippocampal theta rhythm synchronization during fear memory retrieval. *Science* **301**, 846-850 (2003).

13. Clark, K. B., Krahl, S. E., Smith, D. C. & Jensen, R. A. Post-training unilateral vagal stimulation enhances retention performance in the rat. *Neurobiol Learn Mem* **63**, 213-216 (1995).

14. Clark, K. B., Naritoku, D. K., Smith, D. C., Browning, R. A. & Jensen, R. A. Enhanced recognition memory following vagus nerve stimulation in human subjects. *Nat Neurosci* **2**, 94-98 (1999).

15. Gire, D. H., Whitesell, J. D., Doucette, W., & Restrepo, D. Information for decision-making and stimulus identification is multiplexed in sensory cortex. *Nat neurosci*, **16**, 991-993 (2013).

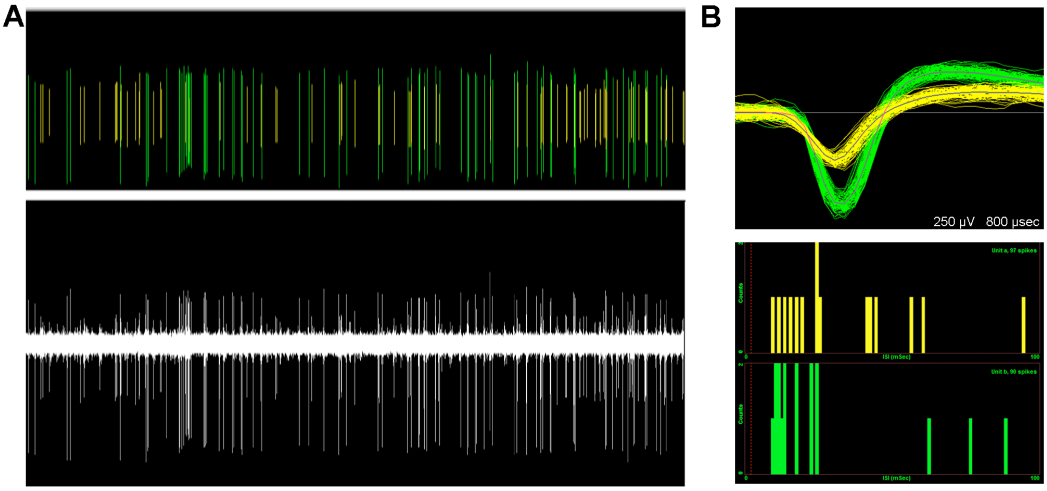

Supplementary Figure 1. Example of two units sorted from one microwire implanted in the ACC. (a) 30 s raw data showing spike firing on one channel in the ACC of an intact rat. The upper panel shows 800µs of waveforms from each spike in yellow color (unit a) and green color (unit b). The bottom panel shows continuous spike signals (band-pass filter 300 Hz - 5 kHz, 4-pole Bessel). (b) Superimposed waveforms (800µs) of the spikes of the two units. The two single-units were well identified according to their respective spike templates (upper panel) and inter spike interval histograms (bottom panel). Each unit exhibited a clearly recognizable refractory period (>2ms) in its ISI histogram.

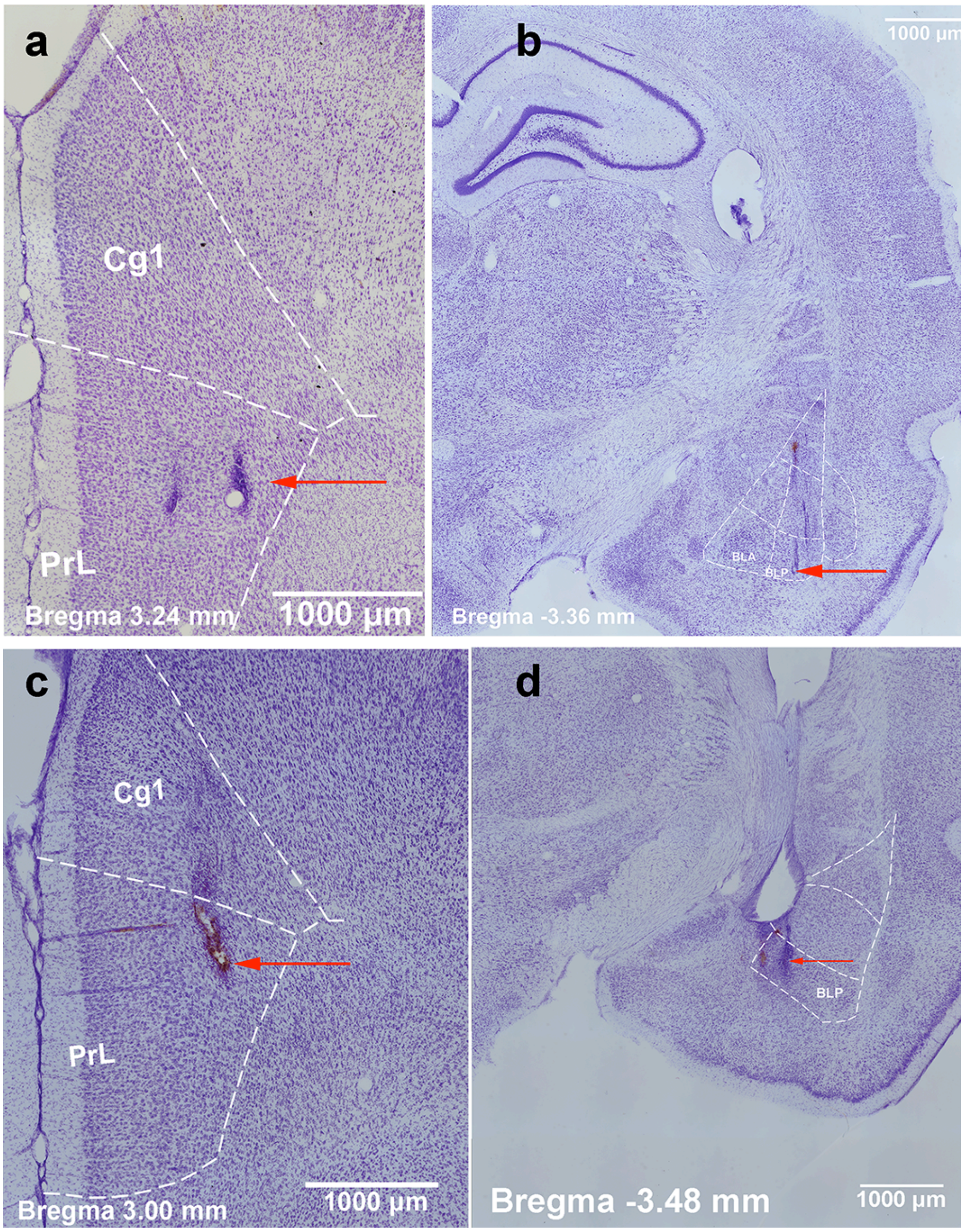

Supplementary Figure 2. Verification of the placement of the recording electrodes. (a, b) The correct placements of the recording electrodes in the ACC (a) and BLA (b) of rat #32 were confirmed by cresyl violet staining of the coronal brain sections. Arrows point to lesions at the end of the electrode tracks. (c, d) The placements of the recording electrodes in rat #33. Cg1, cingulate cortex, area 1; PrL, prelimbic cortex;BLA, basolateral amygdaloid nucleus, anterior; BLP, basolateral amygdaloid nucleus, posterior.

Table 1. Raw percentage of advantageous choice (choice(C+D)/(A+B+C+D) × 100%) during RGT testing session for all rats individually.

Group		Rat #	Percentage of advantageous choice (40-60 min)
Control	Good decision-maker	1	99.50
		4	90.48
		5	75.72
		8	94.30
		9	99.66
		11	79.69
		13	96.10
		14	76.82
		15	83.76
		16	98.89
		20	98.98
		22	100.00
		23	83.48
		25	80.42
		27	77.69
		28	73.80
	undecided decision-maker	2	51.65
		3	36.42
		12	63.63
		17	61.66
		18	45.62

		**24**	**41.75**
		**26**	**44.91**
	**poor decision-maker**	**6**	**26.45**
		**7**	**19.39**
		**10**	**25.58**
		**19**	**19.05**
		**21**	**21.72**
**VNS**	**Good decision-maker**	**1**	**76.37**
		**2**	**98.65**
		**3**	**91.67**
		**4**	**87.77**
		**5**	**99.32**
		**6**	**90.27**
		**8**	**79.26**
		**9**	**98.94**
		**10**	**75.97**
		**11**	**96.38**
		**12**	**100.00**
		**13**	**81.42**
		**14**	**78.91**
		**15**	**79.65**
		**16**	**88.88**
		**18**	**78.98**
		**20**	**79.84**
		**22**	**84.00**

	**23**	**80.95**
	**24**	**76.73**
	**26**	**82.86**
	**27**	**84.48**
	**28**	**92.52**
	**29**	**84.33**
	**31**	**76.94**
**Electrophysiological record**	**32**	**71.31**
**Electrophysiological record**	**33**	**84.43**
**Electrophysiological record**	**34**	**77.25**
**Electrophysiological record**	**35**	**85.24**
**Electrophysiological record**	**36**	**93.21**
**Electrophysiological record**	**37**	**79.01**
**undecided decision-maker**	**7**	**40.23**
	**19**	**52.40**
	**25**	**43.64**
**poor decision-maker**	**17**	**12.10**
	**21**	**22.65**
	**30**	**19.59**
